# Genetic overlap between type 2 diabetes and major depressive disorder identified by bioinformatics analysis

**DOI:** 10.18632/oncotarget.8202

**Published:** 2016-03-19

**Authors:** Hong-Fang Ji, Qi-Shuai Zhuang, Liang Shen

**Affiliations:** ^1^ Shandong Provincial Research Center for Bioinformatic Engineering and Technique, School of Life Sciences, Shandong University of Technology, Zibo, P. R. China

**Keywords:** type 2 diabetes, major depressive disorder, single nucleotide polymorphisms, bioinformatics, Gerotarget

## Abstract

Our study investigated the shared genetic etiology underlying type 2 diabetes (T2D) and major depressive disorder (MDD) by analyzing large-scale genome wide association studies statistics. A total of 496 shared SNPs associated with both T2D and MDD were identified at *p*-value ≤ 1.0E-07. Functional enrichment analysis showed that the enriched pathways pertained to immune responses (Fc gamma R-mediated phagocytosis, T cell and B cell receptors signaling), cell signaling (MAPK, Wnt signaling), lipid metabolism, and cancer associated pathways. The findings will have potential implications for future interventional studies of the two diseases.

## INTRODUCTION

Type 2 diabetes (T2D) and depression are prevalent chronic diseases and have been serious public health burdens around the world. The comorbidity between T2D and depression is proven by a series of epidemiological evidence [1-3]. Many studies indicate that the associations between T2D and depression are bidirectional and the presence of T2D increases the risk of depression, and vice versa [4-6].

Despite improved understanding of shared origins of T2D and depression has been gained in recent years [7-10], more effort is needed to identify the shared genetic etiology underlying the two diseases. A wealth of large-scale genome wide association studies (GWAS) about T2D and major depressive disorder (MDD) have been produced in the past few years. These GWAS data provide opportunity to investigate the shared genetic etiology of the two diseases. In this study, we performed a bioinformatics analysis on single nucleotide polymorphisms (SNPs) for T2D and MDD on the basis of GWAS meta-analysis data. The overlapped SNPs for T2D and MDD were identified and functional enrichment analysis was then performed. The findings will benefit future mechanistic and interventional studies for both diseases.

## RESULTS

During the identification of SNPs that are associated with MDD or T2D on the basis of independent DIAbetes Genetics Replication And Meta-analysis (DIAGRAM) or Psychiatric Genomics Consortium meta-GWAS statistics, multiple GWAS genetic association *p*-values ranging from 1.0E-10 to 1.0E-06 were set as cutoff criteria. The results were shown in Table 1. It can be seen that when setting at *p*-value of 1.0E-06 as cutoff criteria, the number of identified SNPs is comparable. With improved *p*-values, the number of identified SNPs associated with T2D is significantly larger than MDD as shown in Table 1. Using *p*-value threshold at 1.0E-10, we identified 2496 and 64 SNPs associated with T2D and MDD, respectively (Table 1).

**Table 1:** Number of SNPs associated with T2D or MDD and overlapped SNPs associated with both diseases with different p-values as cutoff criteria

*P*-value threshold	Number of SNPS		Number of overlapped SNPs
	T2D	MDD	
1.0E-10	2496	64	0
1.0E-09	4445	505	1
1.0E-08	11280	3943	10
1.0E-07	42271	27410	496
1.0E-06	127905	123041	3966

Through overlapping the identified SNPs associated with T2D or MDD, we obtained the number of overlapped SNPs associated with both diseases with different *p*-values as cutoff criteria. As shown in Table 1, when setting the threshold at *p*-value ≤ 1.0E-09 and ≤ 1.0E-08, there were 1 and 10 overlapped SNPs between T2D and MDD, respectively. When setting the threshold at *p*-value ≤ 1.0E-07, 496 SNPs were identified to be associated with both diseases, which was significantly larger than random chance (overlapped *p*-value of 5.32E-08 and odds ratio = 1.08).

Among the overlapped 496 SNPs, there were 216 SNPs with annotated genes. The functional enrichment analysis of these genes was performed by using the Database for Annotation, Visualization and Integrated Discovery (DAVID) v6.7 [14] to conduct Kyoto Encyclopedia of Genes and Genomes (KEGG) pathway and GO term enrichment analysis. For each of these KEGG pathways, information regarding pathway size, the total number of genes in the pathway, the Benjamini-corrected *p*-value, and the annotated genes were provided in Table 2. We obtained significant enrichment of the annotated genes in 61 KEGG pathways. Notably, we observed that selected enriched KEGG pathways listed in Table 2 pertain to immune responses (Fc gamma R-mediated phagocytosis, T cell receptor signaling, B cell receptor signaling pathway), cell signaling (MAPK, Wnt signaling), lipid metabolism, as well as several cancer associated pathways.

**Table 2:** Overlapped pathways identified in T2D and MDD

Term	Total pathway size	Number of genes modulated by overlapped SNPs	Benjamini-corrected *p*-value	Genes
ECM-receptor interaction	2	2	1.36E-05	HSPG2, COL4A2
Glycerolipid metabolism	8	3	5.46E-05	PPAP2B, DGKB, LIPC
Glycerophospholipid metabolism	8	3	5.46E-05	PPAP2B, DGKB, ACHE
Ether lipid metabolism	2	1	1.36E-05	PPAP2B
Sphingolipid metabolism	2	1	1.36E-05	PPAP2B
Fc gamma R-mediated phagocytosis	3	2	2.05E-05	PPAP2B DNM3
Cytokine-cytokine receptor interaction	4	4	2.73E-05	LEPR, TGFB2, IL2RA, CCL11
Neuroactive ligand-receptor interaction	6	6	4.09E-05	LEPR, PTGER3, GABRB1, GRM8, LPAR2, GRIK1
Jak-STAT signaling pathway	3	3	2.05E-05	LEPR, SPRED2, IL2RA
Adipocytokine signaling pathway	2	2	1.36E-05	LEPR, PPARGC1A
Calcium signaling pathway	5	4	3.41E-05	PTGER3, SLC8A1, CACNA1C, ITPR2
Cell adhesion molecules	3	2	2.05E-05	NEGR1, CNTN1
Wnt signaling pathway	7	6	4.77E-05	VANGL1, PRICKLE2, CTBP2, SMAD3, NFATC3, NFATC2
Dorso-ventral axis formation	2	2	1.36E-05	NOTCH2, ETV6
Notch signaling pathway	2	2	1.36E-05	NOTCH2, CTBP2
Endocytosis	4	4	2.73E-05	DNM3, IL2RA, USP8, GIT1
MAPK signaling pathway	6	6	4.09E-05	TGFB2, MRAS, MAP4K2, CACNA1C, FGF14, NFATC2
Cell cycle	5	3	3.41E-05	TGFB2, E2F3, SMAD3
TGF-beta signaling pathway	3	2	2.05E-05	TGFB2, SMAD3
Pathways in cancer	11	9	7.5E-05	TGFB2, PPARG, E2F3, CTBP2, IGF1, FGF14, COL4A2, SMAD3, DCC
Colorectal cancer	4	3	2.73E-05	TGFB2, SMAD3, DCC
Pancreatic cancer	5	3	3.41E-05	TGFB2, E2F3, SMAD3
Chronic myeloid leukemia	6	4	4.09E-05	TGFB2, E2F3, CTBP2, SMAD3
Hypertrophic cardiomyopathy	7	6	4.77E-05	TGFB2, SLC8A1, TTN, ACTB, CACNA1C, IGF1
Dilated cardiomyopathy	7	6	4.77E-05	TGFB2, SLC8A1, TTN, ACTB, CACNA1C, IGF1
O-Glycan biosynthesis	2	2	1.36E-05	GALNT2, C1GALT1
Cardiac muscle contraction	3	2	2.05E-05	SLC8A1, CACNA1C
Arrhythmogenic right ventricular cardiomyopathy	5	4	3.41E-05	SLC8A1, ACTB, CACNA1C, PKP2
Ubiquitin mediated proteolysis	2	2	1.36E-05	FANCL, UBE2R2
PPAR signaling pathway	2	2	1.36E-05	PPARG, EHHADH
Huntington's disease	5	4	3.41E-05	PPARG, DNAH1, PPARGC1A, CREB5
Valine, leucine and isoleucine biosynthesis	2	2	1.36E-05	LARS2, EHHADH
Aminoacyl-tRNA biosynthesis	2	2	1.36E-05	LARS2, CARS
Axon guidance	6	6	4.09E-05	ROBO2, UNC5C, ABLIM1, NFATC3, DCC, NFATC2
Tight junction	3	3	2.05E-05	MRAS, ACTB, SYMPK
Regulation of actin cytoskeleton	4	4	2.73E-05	MRAS, ACTB, FGF14, GIT1
Insulin signaling pathway	2	2	1.36E-05	PPARGC1A, GCK
Lysosome	3	3	2.05E-05	MANBA, IGF2R, CTSZ
Glioma	2	2	1.36E-05	E2F3, IGF1
Prostate cancer	3	3	2.05E-05	E2F3, CREB5, IGF1
Melanoma	4	3	2.73E-05	E2F3, IGF1, FGF14
Bladder cancer	2	1	1.36E-05	E2F3
Small cell lung cancer	3	2	2.05E-05	E2F3, COL4A2
Non-small cell lung cancer	2	1	1.36E-05	E2F3
Chondroitin sulfate biosynthesis	2	2	1.36E-05	DSE, CSGALNACT1
Focal adhesion	3	3	2.05E-05	ACTB, IGF1, COL4A2
Adherens junction	4	3	2.73E-05	ACTB, PTPRB, SMAD3
Vibrio cholerae infection	5	2	3.41E-05	ACTB, KCNQ1
Phosphatidylinositol signaling system	6	2	4.09E-05	DGKB, ITPR2
Type II diabetes mellitus	2	2	1.36E-05	GCK, CACNA1C
Maturity onset diabetes of the young	3	3	2.05E-05	GCK, HNF1A, HNF4A
Vascular smooth muscle contraction	2	2	1.36E-05	CACNA1C, ITPR2
Long-term potentiation	2	2	1.36E-05	CACNA1C, ITPR2
GnRH signaling pathway	2	2	1.36E-05	CACNA1C, ITPR2
Alzheimer's disease	2	2	1.36E-05	CACNA1C, ITPR2
Oocyte meiosis	2	2	1.36E-05	ITPR2, IGF1
Long-term depression	2	2	1.36E-05	ITPR2, IGF1
VEGF signaling pathway	2	2	1.36E-05	NFATC3, NFATC2
Natural killer cell mediated cytotoxicity	2	2	1.36E-05	NFATC3, NFATC2
T cell receptor signaling pathway	2	2	1.36E-05	NFATC3, NFATC2
B cell receptor signaling pathway	2	2	1.36E-05	NFATC3, NFATC2

## DISCUSSION

Based on the available meta-GWAS statistic for T2D and MDD, we performed a bioinformatics analysis to explore the shared genetic etiology underlying the two diseases. When setting the threshold at *p*-value ≤ 1.0E-07, 496 overlapped SNPs were identified to be associated with both T2D and MDD. Further functional enrichment analysis observed 61 KEGG pathways, including immune responses (Fc gamma R-mediated phagocytosis, T cell and B cell receptors signaling), cell signaling (MAPK, Wnt signaling), lipid metabolism, as well as several cancer associated pathways.

The findings gained supports from previous studies. First, abnormality in the immune and inflammation systems has been reported to be involved in the pathogenesis of both T2D and MDD [11-15]. Several pathways, including Fc gamma R-mediated phagocytosis, T cell and B cell receptors signaling, have been observed (Table 2). Oxidative stress caused by excessive reactive oxygen species (ROS) contribute to the pathogenesis of T2D and MDD. As crucial secondary messengers in signal transduction, ROS may also exert significant effect on inflammatory pathways through MAPK activation [16, 17]. Wnt signal has also been implicated in the development of both T2D and MDD [18-20] In addition, several cancer associated pathways have been found, which may arise from the epidemiologically observed close relationships between diabetes or depression and many types of cancer [21-23].

There are some limitations in our study. First, the functional enrichment analysis of the shared genes of T2D and MDD is based on the accuracy and completeness of KEGG database. Thus, some genes possessing impacts on both diseases but not annotated in KEGG databases are not included. Second, although we employed two most comprehensive large-scale meta-GWAS statistics for T2D and MDD respectively, other GWAS not considered may potentially affect the results.

In summary, through bioinformatics analysis of two most comprehensive large-scale meta-GWAS statistic of T2D and MDD, we identified the overlapped SNPs and performed functional enrichment pathway of the annotated genes. The findings tentatively support the disease concordance between T2D and MDD indicated by epidemiological studies, and also have potential implications for future therapeutic strategies for the two diseases.

## MATERIALS AND METHODS

The identification of SNPs associated with T2D risk was based on the meta-GWAS statistics from the DIAGRAM consortium study, which was generated from a meta-analysis study covering 34,840 cases and 114,981 controls, overwhelmingly of European descent [24]. These T2D statistics provide more than 2 million SNPs on the basis of the calculation using HapMap project. The identification of SNPs associated with MDD was based on the meta-GWAS statistics from the Psychiatric Genomics Consortium study, the largest and most comprehensively genome-wide analysis of MDD yet conducted [25]. This study investigated more than 1.2 million autosomal and X chromosome SNPs in 18759 independent and unrelated subjects of recent European ancestry (9240 MDD cases and 9519 controls) in the MDD discovery phase. In the MDD replication phase, this study also evaluated 554 SNPs in independent 6,783 MDD cases and 50,695 controls [25].

To identify SNPs significantly associated with diseases, multiple cutoff *p*-value criteria were employed. SNPs identified by GWAS were compared to identify overlapped SNPs between T2D and MDD. As we know, for a given complex diseases, such as T2D and MDD, individual genetic variants may interpret only a very small amount of genetic risk. Thus, with the aim to more comprehensively identify SNPs with small effect sizes, a “relaxed” cutoff genetic association *p*-value of 1.0E-07 was employed as a criterion for identifying SNPs that are associated with risk for both T2D and MDD.

The location and mapped genes of each shared SNPs for T2D and MDD were obtained *via* the Single-Nucleotide Polymorphism database (dbSNP) at the National Center for Biotechnology Information (NCBI). Then, functional enrichment analysis of obtained genes was performed by the DAVID v6.7 [26] to perform KEGG pathway and GO term enrichment analysis. The significance of pathway was calculated by statistical method of hypergeometric distribution, and *P*-value of 0.05 was set as the threshold of significance. Significant pathways and GO terms identified in enrichment analysis were compared between T2D and MDD to investigate shared pathways of these two disorders.
